# Overexpression of the third H-NS paralogue H-NS2 compensates fitness loss in *hns* mutants of the enteroaggregative *Escherichia coli* strain 042

**DOI:** 10.1038/s41598-020-75204-4

**Published:** 2020-10-22

**Authors:** A. Prieto, M. Bernabeu, L. Falgenhauer, T. Chakraborty, M. Hüttener, A. Juárez

**Affiliations:** 1 Department of Genetics, Microbiology and Statistics, University of Barcelona, Barcelona, Spain; 2 Institute of Hygiene and Environmental Medicine, Justus Liebig University Giessen, Schubertstrasse 81, 35392 Giessen, Germany; 3 German Center for Infection Research DZIF, Partner Site Giessen-Marburg-Langen, Campus, Giessen, Germany; 4 Institute of Medical Microbiology, Justus-Liebig University, Schubertstrasse 81, 35392 Giessen, Germany; 5 Institute for Bioengineering of Catalonia (IBEC), The Barcelona Institute of Science and Technology, Barcelona, Spain

**Keywords:** Bacteriology, Microbial genetics

## Abstract

Members of the H-NS protein family play a role both in the chromosome architecture and in the regulation of gene expression in bacteria. The genomes of the enterobacteria encode an H-NS paralogue, the StpA protein. StpA displays specific regulatory properties and provides a molecular backup for H-NS. Some enterobacteria also encode third H-NS paralogues. This is the case of the enteroaggregative *E*. *coli* (EAEC) strain 042, which encodes the *hns*, *stpA* and *hns2* genes. We provide in this paper novel information about the role of the H-NS2 protein in strain 042. A C > T transition in the *hns2* promoter leading to increased H-NS2 expression is readily selected in *hns* mutants. Increased H-NS2 expression partially compensates for H-NS loss. H-NS2 levels are critical for the strain 042 fitness. Under some circumstances, high H-NS2 expression levels dictated by the mutant *hns2* promoter can be deleterious. The selection of T > C revertants or of clones harboring insertional inactivations of the *hns2* gene can then occur. Temperature also plays a relevant role in the H-NS2 regulatory activity. At 37 °C, H-NS2 targets a subset of the H-NS repressed genes contributing to their silencing. When temperature drops to 25 °C, the repressory ability of H-NS2 is significantly reduced. At low temperature, H-NS plays the main repressory role.

## Introduction

Nucleoid-associated proteins of the H-NS family contribute to shaping the chromosome and also regulate gene expression mainly in the enterobacteria and in other Gram-negative microorganisms. The *E*. *coli hns* gene codes for a 15.5 kDa protein which is present at about 20.000 copies per cell^1,2^. H-NS generally acts as a repressor of transcription, silencing the expression of several genes and operons. The structural H-NS features and the nature of the complexes that H-NS forms with DNA have been extensively studied^3–6^. H-NS binds preferentially AT-rich DNA stretches, and it has been proposed to operate as a xenogeneic silencer, avoiding unwanted expression of horizontally acquired genes^7–11^. This protein not only modulates the expression of HGT DNA, but also of core genes^12^. Gene silencing mediated by the H–NS protein responds, among other environmental stimuli, to temperature or osmolarity^13–18^.

The genomes from many proteobacteria encode additional *hns* homologues or paralogues. The StpA protein (suppressor of *td* phenotype A^19^) is the best studied H-NS paralogue. The *E*. *coli* StpA protein is 15.3 kDa in size and it is present at about 3.000 molecules per cell^2^. In *E*. *coli*, *stpA* mutants cultured under standard laboratory conditions do not show a clear phenotype. Taking into account that StpA is overexpressed in *hns* mutants^20^, it has been suggested that one of the roles of this protein is to provide a molecular backup for H-NS in the cell^20,21^. Unlike *E*. *coli*, *stpA* mutants show an altered expression of a significant number of genes in *Salmonella*^22^.

*hns* orthologues are also encoded in plasmids^23^. A well-characterized example is the *hns* orthologue *sfh*, which is encoded in the IncHI1 plasmid pSF-R27. The expression of the Sfh protein in cells harboring plasmid pSF-R27 provides a stealth function, avoiding the fact that plasmid incorporation results in a fitness cost for the bacterial host^24^.

Some pathogenic *E*. *coli* strains can also encode additional *hns* paralogues. The uropathogenic strain 536 encodes a third *hns* paralogue, the *hfp* gene^25^. The main regulatory role of the Hfp protein was found to occur at temperatures outside the host (25 °C). The H-NS paralogues StpA, Sfh and Hfp have been proposed to modulate H-NS activity by forming heteromers with H-NS, which display DNA-binding properties different from those from homomeric H-NS^25–27^. The enteroaggregative *E*. *coli* strain 042 also contains a third H-NS paralogue, termed H-NS2^28^. The H-NS2 protein shows 64.4% identity with the H-NS protein. H-NS2 displays specific expression and regulatory properties. H-NS2 targets at 37 °C only a subset of those genes that are regulated by H-NS, and may help to differentially regulate core and HGT genes by the H-NS cellular pool^28^. We present in this report further information about the biological role of the H-NS2 protein. The H-NS2 expression levels can be modified by a spontaneous reversible C > T transition in the promoter region of the *hns2* gene. In strain 042 *hns* mutants, increased H-NS2 expression partially compensates for H-NS loss when cells grow at low temperature. We also show a differential regulatory role of the H-NS2 protein at 25 °C and 37 °C.

## Results

### Passaging of strain 042 Δ***hns*** derivatives for 10 days at 25 °C generates cultures with increased growth rate

In different enterobacterial strains, loss of H-NS function results in a reduced growth rate. An experimental evolution approach designed to identify secondary mutations compensating for H-NS loss in *Salmonella* showed that, when subculturing Δ*hns* mutants, some of the Δ*hns* cell lineages that increased the growth rate accumulated mutations in the *stpA* gene, generating StpA variants with altered DNA binding and oligomerization properties, resembling those of H-NS^29^. We considered that a similar approach could be performed with strain 042 in order to gain insight into H-NS2 function. We hypothesized that, as it was the case with the *stpA* gene in *Salmonella* Δ*hns* mutants^29^, subcultures of a Δ*hns* derivative of the *E*. *coli* strain 042 would generate clones with increased fitness, which could harbor mutations mapping either in the *hns2* or in the *stpA* genes.

**Figure 1:**
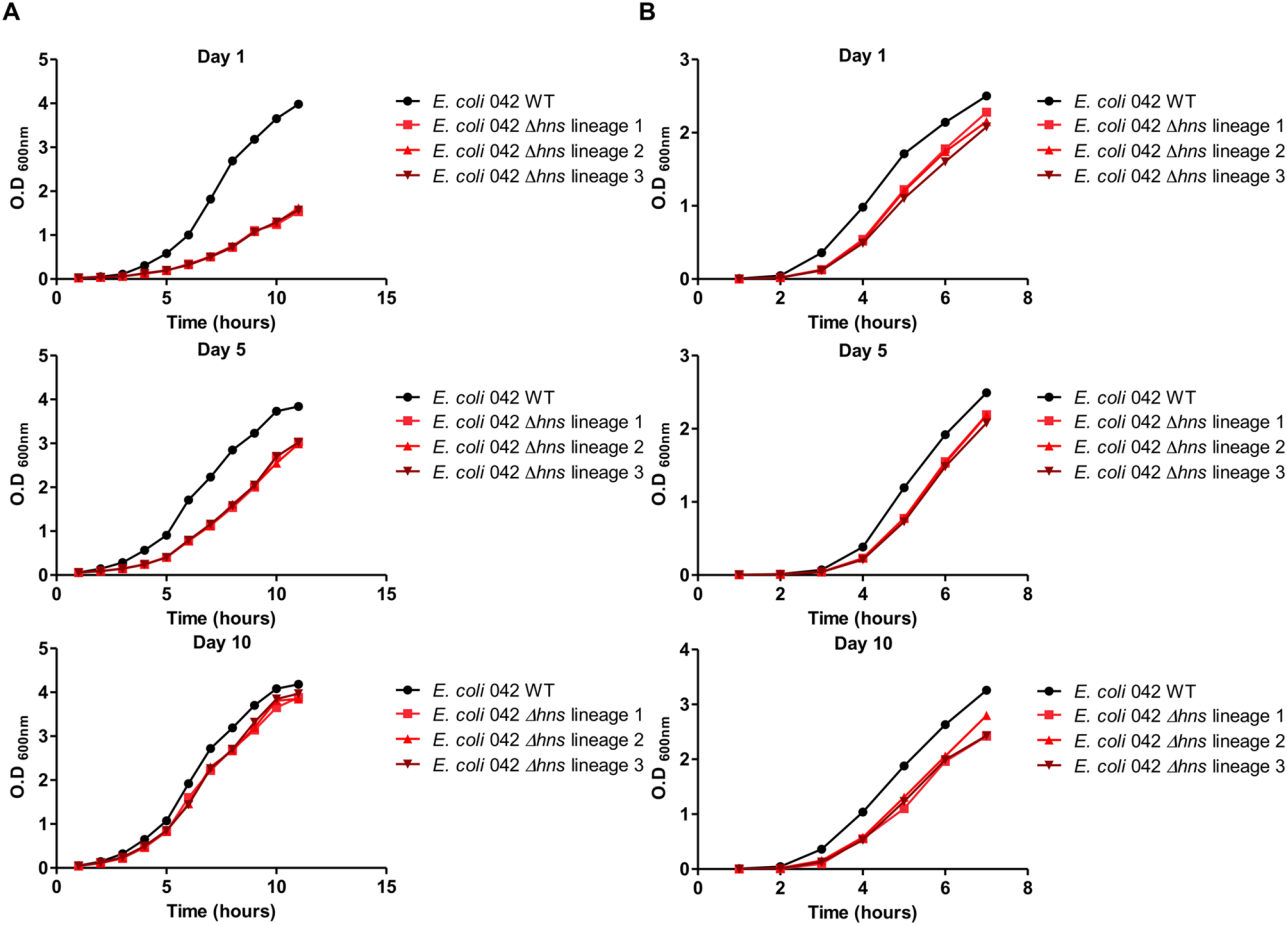
Growth curves of the lineages generated by subculturing one clone of the 042 WT strain and three independent clones of the 042 *hns* derivative, at 25 °C (**A**) and 37 °C (**B**). Growth was determined at days 1 and upon 5 and 10 days of subculturing.

We already checked that Δ*hns* mutants of strain 042 show a reduced growth rate, especially when cells grow at low temperature^28^. For the directed evolution experiment, three independent cultures of strain 042 Δ*hns* were serially passaged alongside one lineage of the WT strain in LB medium and were grown either at 37 °C or at 25 °C for 10 days. Each day aliquots were diluted 1:1.000 for cultures grown at 37 °C, and 1:100 for cultures grown at 25 °C. At days 1, 5 and 10, aliquots were frozen at -80 °C for retrospective analysis of the genetic changes in each lineage over time. At days 1, 5, and 10 growth curves were determined both for the WT and for the different Δ*hns* lineages (Fig. 1A,B). Cultures grown at 37 °C and 25 °C showed different patterns. Although the number of generations of cultures grown at 37 °C was significantly higher than that of cultures grown at 25 °C, Δ*hns* lineages grown at 37 °C did not display significant increases in their growth rates compared to that of the day 1 clone. In contrast, all three Δ*hns* lineages grown at 25 °C displayed significant increases in the growth rate at day 5 and reached values similar to that of the WT clone at day 10 (Table 1A,B).

**Table 1:** Growth rates of the 042 WT strain and the three independent Δ*hns* lineages subcultured both at 25 °C (A) and 37 °C (B).

	*E*. *coli* 042 WT	*E*. *coli* 042 Δ*hns* lineage 1	*E*. *coli* 042 Δ*hns* lineage 2	*E*. *coli* 042 Δ*hns* lineage 3
**(A) Growth rate (µ) h**^**−1**^** 25 °C**				
Day 1	0.51	0.28	0.28	0.28
Day 5	0.50	0.31	0.30	0.32
Day 10	0.53	0.44	0.45	0.46
**(B) Growth rate (µ) h**^**−1**^** 37 °C**				
Day 1	0.79	0.64	0.63	0.61
Day 5	0.80	0.60	0.60	0.58
Day 10	0.83	0.60	0.62	0.60

^(A)^Growth rate data of *E*. *coli* 042 Δ*hns* lineages 1, 2 and 3 from each day was combined and fitted with a quadratic curve (R^2^ = 0.992).

### The three Δ*hns* lineages subcultured at 25 °C contain the same mutation in the *hns2* gene

**Figure 2:**
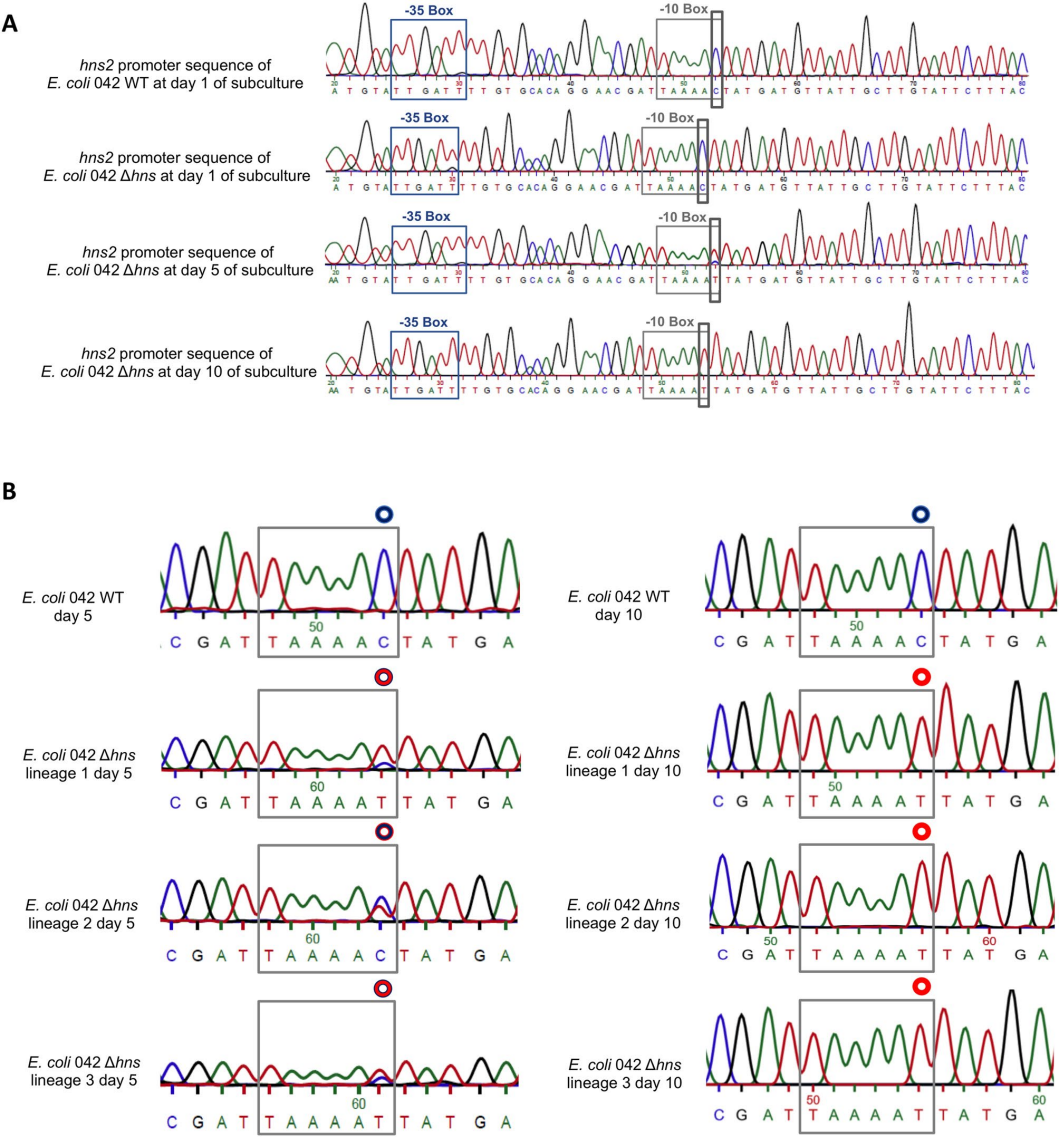
(**A**) Sequencing chromatograms corresponding to the predicted promoter region of the *hns2* gene of strain 042 WT and one of the lineages of the *hns* mutant derivative at different subculture intervals. The -10 sequence of the *hns2* promoter is indicated with a gray box, whereas the -35 sequence is indicated in blue. A black box highlights the C > T transition generated in the *hns2* promoter. (**B**) Sequencing chromatograms corresponding to the -10 sequence of the *hns2* gene promoter region of the 042 WT strain and of the three different lineages of the *hns* mutant derivative upon 5 and 10 days subculture at 25 °C. The blue circle highlights the C present in the WT promoter, whereas the red circle highlights the transition to T.

With the aim to correlate the observed increase in the growth rate in the Δ*hns* subcultures with alterations in either the *hns2* or the *stpA* genes, we used first the 10 days old lineages of the WT and Δ*hns* mutant lineage 1 cultured at 25 °C to amplify by PCR and sequence the *hns2* and *stpA* genes. All three Δ*hns* lineages showed no alterations in the *stpA* gene, but they contained the same mutation in the *hns2* gene: a C > T transition in the predicted promoter region, specifically located in the last position of the -10 box (hereon termed phns2* promoter) (Fig. 2A). We also studied the kinetics of the appearance of the C > T transition at 25 °C in all three Δ*hns* lineages by analyzing the *hns2* promoter sequence in all 5- and 10-days old subcultures (Fig. 2B). In the 5 days old subcultures of clone 1, most of the cells already showed the C > T transition. Subcultures of clones 2 and 3 contained mixtures of cells containing both the WT and mutant *hns2* promoters. In the 10 days old subcultures, the C nucleotide had been totally replaced by T in all three clones (Fig. 2B). Taking into account that the identified mutation occurs within the *hns2* promoter region, we decided to identify the transcriptional start point of the *hns2* gene. We used 5′ RACE to map the + 1 transcriptional start site of the *hns2* gene in strains 042 WT, 042 Δ*hns* and 042 Δhns phns2*. In all three strains, transcription is initiated at a G residue, 7 nucleotides downstream of the -10 box and 41 nucleotides upstream of the *hns2* start codon (Supplementary Fig. S1).

To rule out that other genetic changes out of the *hns2* promoter mutation were underlying the increased growth rate of the Δ*hns* mutant, the genomes of both the WT 042 strain and one of the 10 days old Δ*hns* subculture were sequenced. No other significant genomic alterations (i.e., deletions of specific DNA regions) were detected.

In the *E*. *coli* strain MG1655, in vitro evolution experiments performed with a double *hns stpA* mutant revealed that either point mutations inactivating the sigma factor RpoS or an amplification of ∼40% of the chromosome centered around the origin of replication could partially compensate the physiological alterations caused by the simultaneous loss of H-NS and StpA^30^. We decided to assess whether similar changes could be selected in a 042 double *hns stpA* mutant. To our surprise, the double *hns stpA* mutant showed a similar growth rate than that of the WT strain. We sequenced then the *hns2* gene in the *hns stpA* mutant strain. It contains the same identified C > T transition in the promoter region.

### The identified mutation in the *hns2* promoter increases H-NS2 expression

The likely explanation for the relationship between the C > T transition in the mutant *hns2* promoter and the increased growth rate of the 10 days old subcultures of the Δ*hns* mutants at 25 °C is that the H-NS2 expression is higher in the Δ*hns* clone harboring the phns2* promoter than in the Δ*hns* clone harboring the WT *phns2* promoter. To demonstrate this, we studied both *hns2* transcription and H-NS2 expression in clones harboring respectively the WT *phns2* and mutant phns2* promoters.

**Figure 3:**
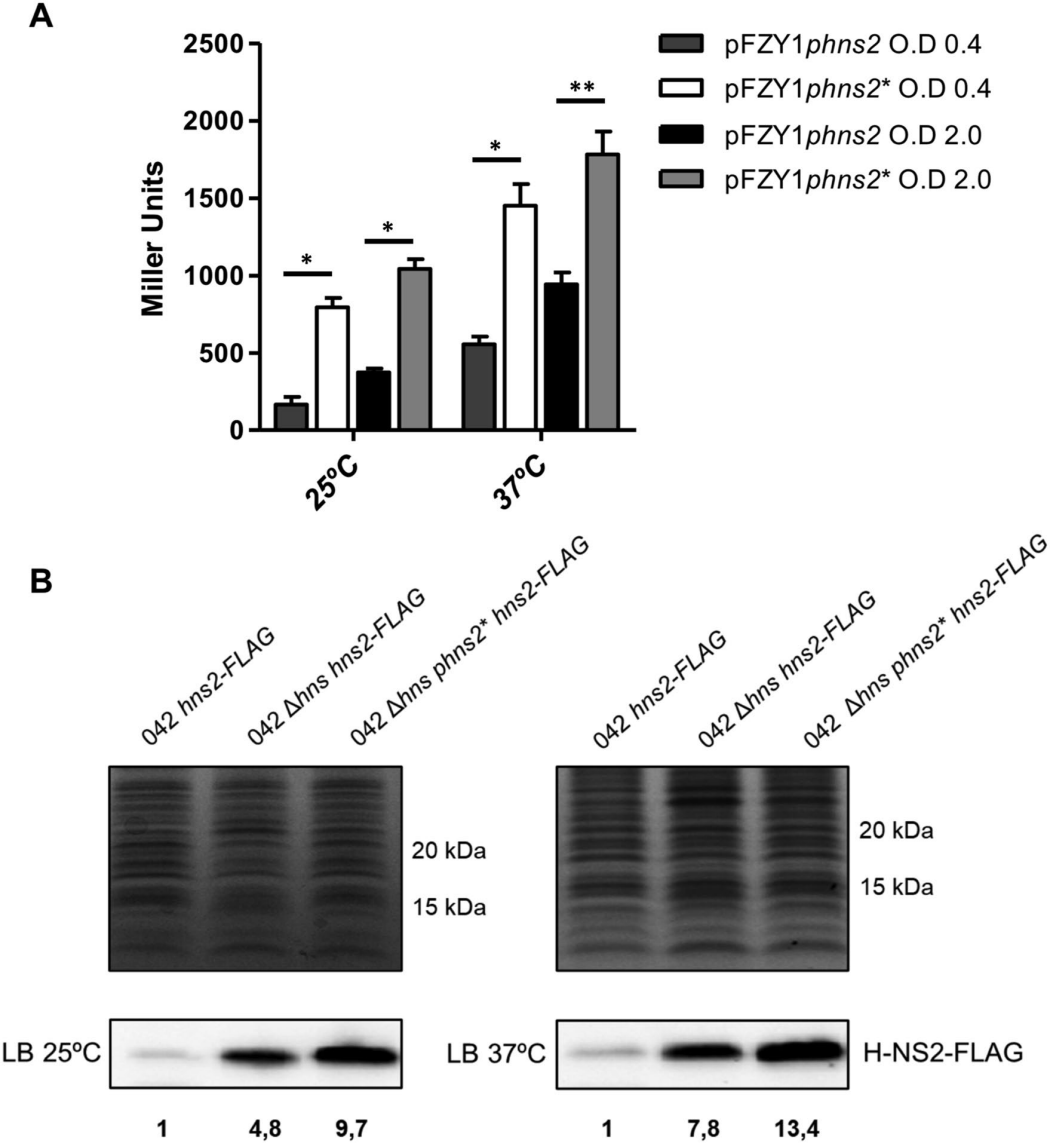
(**A**) Transcription of the *hns2* gene under the control of *phns2* and phns2* promoters. Samples were collected at the exponential (OD_600_ 0.4) and early stationary (OD_600_ 2.0) growth phases. The bar shows the arithmetic mean of three independent experiments and the error bar indicates the standard deviation. Statistical analysis showed a significant difference (*P-value < 0.0001, **P-value < 0.001). (**B**) Expression of the H-NS2 protein under the control of *phns2* and phns2* promoters. Immunodetection of the FLAG-tagged H-NS2 protein with anti-FLAG antibodies, both at 25 °C and 37 °C. Upper figures correspond to total cell extracts stained with Coomassie blue (control of the amount of protein charged). Lower figures show immunodetected H-NS2 protein. Numbers correspond to the relative amount of H-NS2-Flag protein found in the different strains as compared to the WT (set as 1).

To assess the transcriptional activity of the WT and phns2* promoters, they were cloned in the pFZY1 plasmid and transformed in the strain 042 Δ*lacZ*. Samples from cultures of strains 042 Δ*lacZ* (pFZY1*phns2*) and 042 Δ*lacZ* (pFZY1phns2*) grown in LB medium both at 25 °C and at 37 °C were collected at the exponential (OD_600_ 0.4) and the early stationary (OD_600_ 2.0) growth phases, and the β-galactosidase activity was determined (Fig. 3A). As expected, the β-galactosidase activity of cells harboring the phns2* promoter is higher than that of cells harboring the WT *phns2* promoter in all samples analyzed.

To correlate the transcriptional activity dictated by both *phns2* and phns2* promoters with protein expression, a Flag-tag was added to the C-terminal end of the *hns2* gene of strains 042 WT, 042 Δ*hns* and 042 Δhns phns2*. Cultures of strains *hns2-FLAG*, Δ*hns hns2-FLAG* and Δhns phns2^*^ hns2-FLAG were grown both at 37 °C and at 25 °C in LB medium, cell extracts were obtained at an OD_600_ of 2.0 and the H-NS2 protein was detected by Western blotting (Fig. 3B). As previously reported^28^, H-NS2 is overexpressed in an Δ*hns* mutant. As expected, H-NS2 levels are higher in strain Δhns phns2* than in strain 042 Δ*hns*.

We also analyzed the phns2* sequence by using the BPROM software. This analysis showed that a recognition sequence for the CpxR transcription factor is generated in the phns2* promoter (Supplementary Fig. S2). CpxR is the two-component response regulator that is sensitive to phosphorylation by CpxA. This two-component system responds to, among other environmental factors, periplasm and outer membrane stress^31^. We decided to study whether the mutant promoter phns2* was activated by CpxR. To that end, we obtained a Δ*cpxR* derivative of strain 042 Δ*lacZ*. The Δ*lacZ* Δ*cpxR* derivative was transformed with plasmids pZYF1*phns2* and pZYF1phns2*. The presence of the *cpxR* allele did not modify the induction of the transcription of the *lacZ* gene under the control of the phns2* promoter (Supplementary Fig. S3).

### H-NS2 expression levels influence the growth rate of the WT 042 and other *E*.* coli* strains

**Figure 4:**
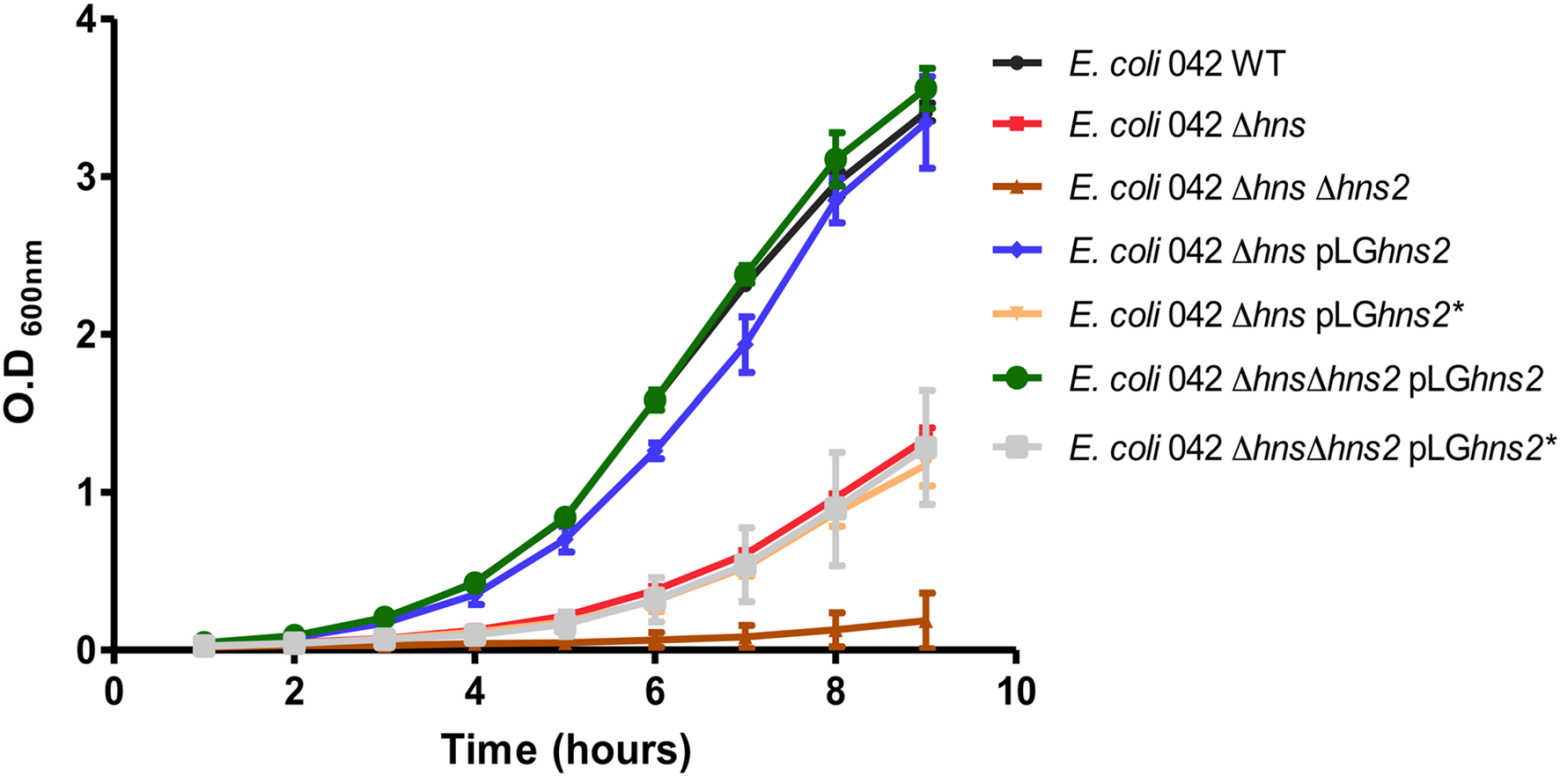
Growth curves of strain 042 WT and its *hns* and *hns hns2* derivatives, both plasmid-free and harboring plasmids pLG*hns2* and pLGhns2*.

To further evaluate whether the H-NS2 expression levels influence the growth rate in different strains, we cloned both the *hns2* and hns2* genes in the vector pLG338-30, generating plasmids pLG*hns2* and pLGhns2*. We transformed them in strains 042 Δ*hns* and 042 Δ*hns* Δ*hns2* and analyzed the growth curve of the transformants in LB medium at 25 °C. Strains 042 Δ*hns* (pLG*hns2*) and 042 Δ*hns* Δ*hns2* (pLG*hns2*) showed a growth rate similar to that of the WT strain (Fig. 4). This suggests that pLG*hns2*-dependent H-NS2 expression levels compensate either for H-NS or H-NS/H-NS2 loss. In contrast, the growth rate of strain 042 Δ*hns* (pLGhns2*) was lower than that of the Δ*hns* mutant and the growth rate of strain Δ*hns* Δ*hns2* (pLGhns2*) was higher than that of strain Δ*hns* Δ*hns2*, but significantly lower than that of the WT strain. This shows that expression of H-NS2 dictated by plasmid pLGhns2* is not compensating for H-NS loss. To clarify this, we amplified by PCR and sequenced the *hns2* gene from strains 042 Δ*hns* (pLG*hns2*) and 042 Δ*hns* (pLGhns2*). PCR amplification of the *hns2* gene in the former strain generated a single band, and the *hns2* sequence obtained from strain 042 Δ*hns* (pLG*hns2*) corresponded to the WT *hns2* gene. In contrast, PCR amplification of the *hns2* gene from strain 042 Δ*hns* (pLGhns2*) generated two bands (Supplementary Fig. S4A). The size of one of them corresponded to the *hns2* gene (likely resulting from the amplification of the chromosomal copy). Sequencing of the higher molecular mass amplicon showed that an *IS5* element is inserted in the coding sequence of the hns2* gene, thus disrupting the *hns2* coding sequence (Supplementary Fig. S4B). This explains why H-NS2 expression dictated by plasmid pLGhns2* does not restore the WT growth rate in strain 042 Δ*hns* (pLGhns2*).

To further confirm that expression of H-NS2 dictated by plasmid pLGhns2* can be unwanted under some circumstances, we transformed both plasmids in strain XL1blue, and amplified by PCR and sequenced the *hns2* gene. Again, the *hns2* sequence of plasmid pLG*hns2* corresponded to WT *hns2*, and that from plasmid pLGhns2* was disrupted by an *IS5* element. In this context it is worth mentioning here that, when cloning the hns2* gene in the pFZY1 plasmid for the *hns2* transcriptional studies, an intermediate step previous to the transformation of the recombinant plasmid in strain 042 is to transform the construct in an intermediate *E*. *coli* host. We tried as intermediate hosts three different strains: DH5α, XL1Blue and MG1655. In the former two strains, it was impossible to recover the pFZY1 plasmid harboring the hns2* gene. All isolates contained plasmids with the WT gene (i.e., the C > T transition had reverted). However, the construct pFZY1hns2* was stable in strain MG1655, and we could hence isolate plasmid from this strain and transform in strain 042 for the transcriptional analysis.

### Temperature and role of the H-NS and H-NS2 proteins modulating gene expression in strain 042

Temperature-dependent regulation of gene expression is a hallmark of the H-NS protein^32^. We showed previously that, at 37 °C, H-NS2 participates in the regulation of the expression of a subset of the H-NS modulated genes, mainly HGT genes^28^. Taking into account that the relationship between increased H-NS2 levels and enhanced growth rate in the 042 Δ*hns* mutant takes place at 25 °C, we decided to study the regulatory role of both proteins in strain 042 at this temperature. To that end, we used RNA-seq to obtain the transcriptomes of strains 042 WT, 042 Δ*hns* and 042 Δ*hns2* at 25 °C. Supplementary figure S5A,B show the functional categories of up-and downregulated genes in strains Δ*hns* and Δ*hns2* when compared to the WT strain. Pathogenesis and bacterial metabolism are the functional categories that include a significant percentage of the genes that are upregulated in both Δ*hns* and Δ*hns2* mutants. Those most upregulated genes in the Δ*hns* and Δ*hns2* mutants at low temperature were identified and compared with the previously identified upregulated genes when cultures were grown at 37°C^28^. Table 2A,B compare those most upregulated genes in the Δ*hns* genetic background at 25 °C and 37 °C. Table 3A,B compare those most upregulated genes in the Δ*hns2* genetic background at these two temperatures. The impact of the growth temperature on the regulatory role of H-NS and H-NS2 proteins is different. For the Δ*hns* mutant, it is apparent that the sets of upregulated genes at 25 °C and 37 °C are similar, although the silencing role of H-NS is much stronger at low than at high temperature (deregulated genes show a much higher foldchange at 25 °C than at 37 °C). In contrast, the effect of the *hns2* allele at low temperature is different to the effect observed at high temperature. The upregulated genes at 37 °C in the Δ*hns2* mutant (which are also upregulated in the Δ*hns* mutant^28^) are not upregulated at 25 °C. At this later temperature, the set of genes showing high fold change values includes mainly components of the nitrate reductase system. Other genes showing moderate foldchange values belong as well to the bacterial metabolism category. They are also upregulated in the *hns2* mutant at high temperature, although exhibiting modest foldchange values. These genes are also upregulated in the *hns* mutant both at 25 °C and 37 °C.

**Table 2:** (A) List of the genes showing the highest fold change values in the 042 Δ*hns* mutant cultured at 25 °C (left column). The corresponding fold change values obtained for the same genes when the strain was grown at 37 °C are shown in the right column. (B) List of genes showing the highest fold change values in the 042 Δ*hns* mutant grown at 37 °C (left column). The corresponding fold change values obtained for the same genes when the strain has been grown at 25 °C is shown in the right column.

NCBI identifier (locus tag)	Fold change	
	Δ*hns* 25 °C	Δ*hns* 37 °C
**(A)**		
EC042_RS07290 (general stress protein)	1137.5	9.3
EC042_RS07280 (ferritin-like domain-containing protein)	736.5	8.8
EC042_RS00760 (fimbrial protein)	718.8	4.3
EC042_RS12270 (colanic acid biosynthesis acetyltransfer WcaF)	581.7	9.8
EC042_RS12260 (GDP-L-fucose synthase WcaG)	505.7	5.4
EC042_RS24025 (cadaverine/lysine antiporter CadB)	492.4	7.0
EC042_RS20090 (hypothetical protein membrane protein)	438.8	14.0
EC042_RS12275 (colanic acid biosynthesis glycosyltransferase WcaE)	427.6	7.4
EC042_RS08585 (glutamate decarboxylase)	371.1	4.3
EC042_RS17480 (thymidylate kinase)	364.7	4.7
EC042_RS12255 (GDP-mannose mannosyl hydrolase)	352.9	6.1
EC042_RS12280 (putative colanic acid polymerase WcaD)	332.5	6.6
EC042_RS03510 (DUF1266 domain-containing protein)	308.5	4.0
EC042_RS01730 (putative fimbrial transcriptional regulator MatA)	306.4	22.7
EC042_RS12250 (colanic acid biosynthesis glycosyltransferase WcaI)	294.4	6.8
EC042_RS20240 (MgtC/SapB family protein)	292.1	7.3
EC042_RS01795 (reactive chlorine species resistance protein RclC)	289.9	7.0
EC042_RS12265 (GDP-mannose 4,6-dehydratase)	288.4	10.5
EC042_RS08580 (glutamate/gamma-aminobutyrateantiporter GadC)	273.3	34.7
EC042_RS03225 (LPS O-antigen length regulator)	263.4	6.1
EC042_RS01800 (DUF1471 domain-containing protein)	260.2	6.6
EC042_RS10355 (GGDEF domain-containing protein)	249.9	7.5
EC042_RS20290 (glutamate decarboxylase alpha subunit GadA)	247.8	37.6
EC042_RS07285 (ferritin-like domain-containing protein)	245.6	8.0
EC042_RS22730 (hypothetical protein)	243.5	3.4
EC042_RS13790 (fimbrial protein)	235.5	4.2
EC042_RS08620 (acid stress response transcriptional regulator YdeO)	226.4	7.1
EC042_RS12365 (DUF2314 domain-containing protein)	223.0	6.4
EC042_RS17785 (fimbrial-like protein)	222.5	3.4
EC042_RS20250 (putative periplasmic acid stress chaperone HdeA)	222.0	26.1
EC042_RS08380 (type VI secretion system PAAR protein)	221.3	5.6
EC042_RS20255 (putative acid resistance protein HdeD)	219.3	17.8
EC042_RS08045 (porin OmpN)	211.6	4.8
EC042_RS22645 (porin OmpL)	205.7	7.1

NCBI identifier (locus tag)	Fold change	
	Δ*hns* 37 °C	Δ*hns* 25 °C
**(B)**		
EC042_RS08585 (glutamate decarboxylase beta subunit GadB)	43.0	371.1
EC042_RS20290 (glutamate decarboxylase alpha subunit GadA)	37.6	247.8
EC042_RS08580 (glutamate/gamma-aminobutyrate antiporter GadC)	34.7	273.2
EC042_RS01725 (putative fimbrial protein MatB)	33.3	128.5
EC042_RS20250 (putative periplasmic acid stress chaperone HdeA)	26.1	–
EC042_RS01730 (putative fimbrial transcriptional regulator MatA)	22.7	30.6
EC042_RS05780 (tRNA-Ser)	19.3	–
EC042_RS15335 (DNA-binding protein StpA)	18.6	72.1
EC042_RS20255 (putative acid resistance protein HdeD)	17.8	219.3
EC042_RS20260 (transcriptional regulator GadE)	16.6	154.7
EC042_RS06765 (tRNA-Tyr)	16.3	–
EC042_RS13160 (hypothetical protein)	15.7	–
EC042_RS21295 (CesD/SycD/LcrH family type III secretion system chaperone)	15.6	22.9
EC042_RS01715 (putative fimbrial outer membrane usher protein)	15.1	70.0
EC042_RS20245 (acid-activated periplasmic chaperone HdeB)	15.1	187.9
EC042_RS06770 (tRNA-Tyr)	14.8	–
EC042_RS04580 (hypothetical protein)	14.5	13.5
EC042_RS01720 (putative fimbrial protein)	14.2	157.9
EC042_RS20090 (hypothetical protein membrane protein)	14.0	438.8
EC042_RS07750 (type I toxin-antitoxin Iar)	13.9	–
EC042_RS01270 (hypothetical protein)	13.6	88.2
EC042_RS24770 (tRNA-Gly)	13.5	–
EC042_RS09275 (hypothetical protein)	13.3	–
EC042_RS01065 (tRNA-Ala)	12.8	–
EC042_RS18940 (tRNA-Ala)	12.8	–
EC042_RS26795 (hypothetical protein)	12.2	–
EC042_RS16270 (hypothetical protein)	12.2	30.3
EC042_RS11015 (tRNA-Leu)	12.1	–
EC042_RS11620 (tRNA-Arg)	12.0	–

**Table 3:** (A) List of the genes showing the highest fold change values in the 042 Δ*hns2* mutant grown at 25 °C (left column). The corresponding fold change values obtained for the same genes when the strain was grown at 37 °C is shown in the right column. These genes are not upregulated in an *hns* mutant. (B) List of genes showing the highest fold change values in the 042 Δ*hns2* mutant cultured at 37 °C (left column). The corresponding fold change values obtained for the same genes when the strain has been grown at 25 °C is shown in the right column. All these genes are also upregulated in an *hns* mutant.

NCBI identifier (locus tag)	Fold change	
	Δ*hns*2 25 °C	Δ*hns*2 37 °C
**(A)**		
EC042_RS06740 (nitrate reductase subunit alpha NarZ)	44.9	2.9
EC042_RS06735 (nitrate transporter NarK)	23.6	2.6
EC042_RS06745 (nitrate reductase subunit beta NarH)	21.0	3.2
EC042_RS06750 (nitrate reductase molybdenum cofactor assembly chaperone NarJ)	13.2	2.4
EC042_RS06755 (respiratory nitrate reductase subunit gamma NarI)	12.8	3.1
EC042_RS03510 (DUF1266 domain-containing protein)	8.2	5.0
EC042_RS10415 (DUF1869 domain-containing protein)	6.7	4.9
EC042_RS16380 (EscR/YscR/HrcR family type III secretion system export apparatus protein)	6.3	2.8
EC042_RS22730 (hypothetical protein)	5.4	5.0
EC042_RS19330 (nitrite reductase large subunit)	4.9	2.9
EC042_RS19990 (nickel ABC transporter)	4.7	3.7
EC042_RS08490 (formate dehydrogenase subunit beta FdxH)	4.6	2.8
EC042_RS03055 (hypothetical protein)	4.5	2.5
EC042_RS08485 (formate dehydrogenase-N subunit alpha FdnG)	4.5	2.1
EC042_RS02000 (galactoside O-acetyltransferase LacA)	4.5	3.2
EC042_RS07770 (hypothetical protein)	4.5	5.9
EC042_RS08480 (sulfate ABC transporter substrate-binding protein)	4.2	–
EC042_RS19335 (nitrite reductase small subunit NirD)	4.0	3.3
EC042_RS28855 (hypothetical protein)	4.0	–
EC042_RS19660 (fimbria/pilus periplasmic chaperone)	3.6	3.9
EC042_RS12365 (yegJ family protein)	3.6	4.1
EC042_RS08380 (type VI secretion system PAAR protein)	3.6	3.0
EC042_RS13110 (ferredoxin-type protein NapF)	3.5	–
EC042_RS08495 (formate dehydrogenase cytochrome b556 subunit)	3.4	2.6
EC042_RS23725 (cytochrome c-552 NrfA)	3.3	–
EC042_RS01960 (2-methylcitrate dehydratase)	3.1	–
EC042_RS28795 (hypothetical protein)	3.1	–

NCBI identifier (locus tag)	Fold change	
	Δ*hns2* 37 °C	Δ*hns2* 25 °C
**(B)**		
EC042_RS05870 (hypothetical protein)	12.1	–
EC042_RS09275 (hypothetical protein)	11.8	–
EC042_RS07145 (hypothetical protein)	11.3	–
EC042_RS26995 (hypothetical protein)	11.3	–
EC042_RS18795 (hypothetical protein)	11.2	–
EC042_RS11295 (hypothetical protein)	10.3	–
EC042_RS13835 (tRNA-Arg)	10.1	–
EC042_RS24055 (antitoxin hypothetical protein)	9.6	–
EC042_RS22745 (DUF3521 domain-containing protein)	9.6	–
EC042_RS03185 (hypothetical protein)	9.3	–
EC042_RS17205 (hypothetical protein)	9.2	–
EC042_RS17210 (hypothetical protein)	9.1	–
EC042_RS03005 (envYporin thermoregulatory protein)	9.0	–
EC042_RS24455 (hypothetical protein)	8.8	–
EC042_RS17185 (putative ATP/GTP-binding protein)	8.7	–
EC042_RS17230 (hypothetical protein)	8.6	–
EC042_RS21255 (putative prophage protein)	8.6	–
EC042_RS25650 (putative RadC-like DNA repair protein)	8.5	–
EC042_RS18375 (hypothetical protein)	8.5	–
EC042_RS17220 (putative DNA repair protein)	8.5	–
EC042_RS11730 (hypothetical protein)	8.4	–
EC042_RS24065 (putative plasmid-like protein)	8.4	–
EC042_RS17215 (putative antirestriction protein)	8.4	–
EC042_RS07390 (hypothetical protein)	8.3	–
EC042_RS12065 (hypothetical protein)	8.3	–
EC042_RS13250 (hypothetical protein)	8.2	–
EC042_RS20520 (putative acetyltransferase)	8.1	–
EC042_RS23195 (hypothetical protein)	8.0	–
EC042_RS17225 (hypothetical protein)	8.0	–

**Figure 5:**
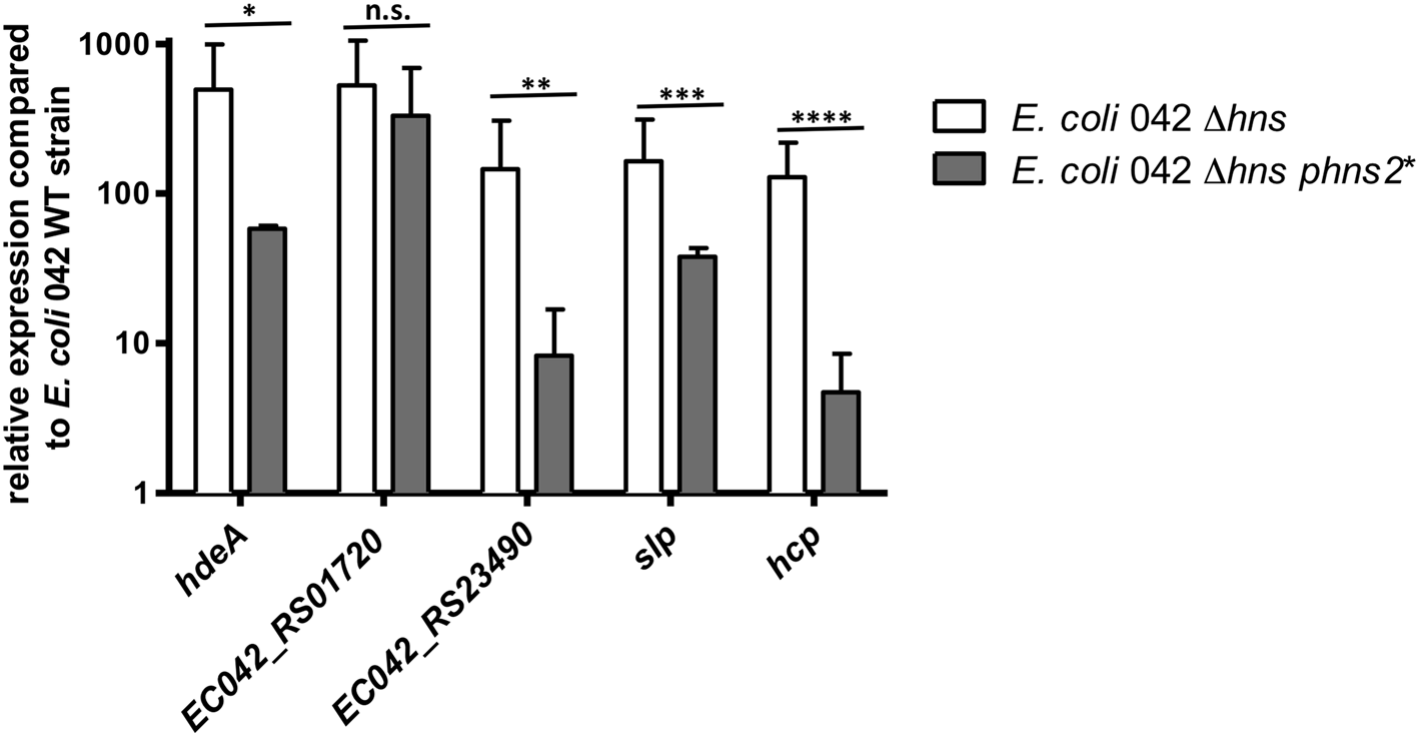
Relative expression (compared to the 042 WT strain) of five selected genes in the *hns* and hns phns2* strains quantified by qRT-PCR in three independent experiments. The bar shows the arithmetic mean of the results of three independent experiments and the error bar indicates the standard deviation. Statistical analysis showed a significant difference (*P-value < 0.0001, n.s., not significant, **P-value < 0.0091, ***P-value < 0.0007, ****P-value < 0.0066).

We also assessed whether, at 25 °C, overexpression of H-NS2 can reduce expression of those genes that are upregulated in an H-NS mutant. To that end, we selected five genes that are overexpressed in a 042 Δ*hns* mutant at 25 °C, but not in an *hns2* mutant under the same culture conditions. We used qRT-PCR to quantitate transcription of these genes both in a 042 Δ*hns* mutant derivative of strain 042 and in one of the 042 Δ*hns* derivatives that contains the C > T transition in the *hns*2 promoter (Fig. 5). When compared to the expression observed in the 042 Δ*hns* mutant, expression in strain 042 Δhns phns2* is significantly lower in four of all five genes studied.

## Discussion

Based on the results of the approach designed in *Salmonella* to identify mutations that increase the growth rate of *hns* mutants^29^, we expected to identify amino acid substitutions either in the StpA or in the H-NS2 sequences that would increase the capacity of any of these protein to more efficiently replace the function of the H-NS protein in the *E*. *coli* strain 042. Nevertheless, this was not the case. Growth of the 042 Δ*hns* mutant at 25 °C readily led to the isolation of mutants mapping in the promoter region of the *hns2* gene. When growing at this temperature, the fact that the different Δ*hns* subcultures exhibiting a growth rate close to that of the WT strain did not harbor mutations in the *stpA* gene but the same C > T transition in the promoter region of the *hns2* gene shows that this latter mutation is a genetic change that occurs at a frequency that, when needed, allows mutants to be readily selected. This was also the case for the double *stpA hns* derivative of strain 042. Rather than accumulating mutations in the *rpoS* gene or suffering amplifications of chromosomal sequences as it was the case for the *E*. *coli* strain MG1655^30^, the above referred mutation in the *hns2* promoter region was selected upon subculturing the strain in the lab.

The C > T transition in the *hns2* promoter increasing the growth rate of the 042 Δ*hns* mutant is likely due to the fact that the resulting mutant promoter is stronger than the WT promoter and dictates an increased H-NS2 expression. A thymine at position -7 in the *hns2* promoter is highly conserved in the TATA box of *E*. *coli* promoters and hence it justifies the increased promoter strength. No other effects (i.e., an altered transcriptional start site of the *hns2* gene or the generation of novel target sequences for transcriptional activators) could be observed.

H-NS2 expression is significantly lower than H-NS expression in the WT strain 042. H-NS represses either directly or indirectly H-NS2 expression^28^. Hence, expression levels of the H-NS2 protein that are higher than those of the WT strain can only be positively selected under some circumstances (i.e., H.NS depletion). In other conditions, abnormally high-level H-NS2 expression can be actually deleterious. Examples are *E*. *coli* strains DH5α and XL1Blue, in which transformation of plasmid pFZY1phns2* generates clones harboring the WT *hns2* promoter (this, in turn, showing that the C > T transition can be reverted), or strains 042 Δ*hns* and XL1blue harboring plasmid pLGhns2*, in which the *hns2* gene is disrupted by the insertion of an *IS* element. This shows that there exists an expression range of H-NS2 which may be beneficial for strain 042 under different conditions but, beyond that range, the effect on cell fitness is detrimental. The analysis of the differential role of the H-NS2 protein at 25 °C and 37 °C also correlates H-NS2 concentration and cell physiology. H-NS2 represses the expression of a subset of the H-NS regulated genes at 37 °C, but not at 25 °C. At low temperature, H-NS2 expression drops^28^ and its role modulating gene expression is reduced. Only few genes show significant foldchange values.

The comparative analysis of the global transcriptomes of strain 042 Δ*hns* at 25 °C and 37 °C is in accordance with the observed different impact of the *hns* mutation on the strain 042 growth rate at both temperatures. The foldchange of H-NS silenced genes at 25 °C is several times higher than that observed at 37 °C. Therefore, a drastically altered expression of several genes in the *hns* mutant growing at 25 °C must significantly impact the cell physiology and the growth rate. As shown in this work, phns2* directed expression of H-NS2 reduces the overexpression of these genes in the *hns* mutant, which in turn must result in an increased growth rate. With respect to the H-NS2 protein, the fact that its expression is lower at 25 °C than at 37°C^28^ is likely underlying the reduced regulatory role that H-NS2 shows at low temperature.

Full induction of H-NS/H-NS2 repressed genes at 37 °C in strain 042 must occur in response to environmental stimuli different to temperature. The likely scenario for several of the H-NS targeted genes is: (i) H-NS strongly represses them at low temperature; (ii) a temperature increase (i.e., transition from 25 °C to 37 °C) alleviates H-NS silencing, enabling a partial expression of these genes. At that temperature (37 °C), both H-NS and H-NS2 contribute to partial gene silencing, and (iii), in response to other stimuli (i.e., osmolarity, pH, anti-silencing factors), H-NS/H-NS2 silencing is completely alleviated.

From the previously published data^28^ and the results presented here at least two functions can be attributed to the H-NS2 protein: to coregulate gene expression with H-NS mainly at temperatures within the host, and to compensate for reduced H-NS (or H-NS/StpA) levels under specific circumstances.

The physiological conditions requiring that H-NS2 compensates for reduced H-NS levels must not be restricted to the occurrence of spontaneous mutations in the *hns* gene. Incorporation of HGT DNA by bacterial cells may result in the resident H-NS protein being titrated out because of targeting incoming AT-rich stretches of HGT DNA. Under these conditions, an increase of H-NS2 expression can avoid fitness loss. As commented above, some plasmids such as pSF-R27 encode an *hns* orthologue, the *sfh* gene. It was shown that expression of the Sfh protein in cells harboring plasmid pSF-R27 provides a stealth function, avoiding the fact that plasmid incorporation reduces host fitness because of resident H-NS protein being titrated out^24^. This function can be also accomplished by the chromosomally encoded H-NS2 protein. Hence, strains that have incorporated the *hns2* gene provide themselves with a powerful regulatory tool that helps H-NS function under different physiological conditions.

In addition to the EAEC strain 042, the H-NS2 protein is expressed in several other pathogenic *E*. *coli* strains and other enterobacterial genera, such as *Salmonella*, *Klebsiella*, *Serratia* or *Citrobacter*. In *E*. *coli*, the vast majority of the strains carrying the *hns2* gene correspond to extraintestinal isolates encoding multiple antibiotic resistance genes in their genomes, such as *Escherichia coli* strain 4/1–1, *Escherichia coli* strain K-15KW01 and *Escherichia coli* strain SaT040. From the EAEC strains whose genome sequence is known, strain 042 is the only one encoding the *hns2* gene. This speaks for *hns2* acquisition being a recent event in this strain, happening upon differentiation of the EAEC pathotype from other *E*. *coli* lineages, H-NS2 comodulates with H-NS several virulence genes in strain 042, including up to 80 pAA genes, which speaks for the relevant regulatory role that this H-NS paralogue plays in strain 042.

## Methods

### Bacterial strains, plasmids and growth conditions

All the bacterial strains and plasmids used in this work are listed in Supplementary Table S1. Oligonucleotides used in this work are listed in Supplementary Table S2.

Bacterial cultures were grown in Luria Broth (LB) medium (tryptone 10 g/l, yeast extract 5 g/l and sodium chloride 10 g/l) at 25 °C or 37 °C with vigorous shaking at 200 rpm (*Innova 3100 water bath shaker*, *New Brunswick Scientific*). When required, the media was supplemented with the antibiotics carbenicillin (Cb) 50 μg/ml, kanamycin (Km) 50 μg/ml, chloramphenicol (Cm) 25 μg/ml, or tetracycline (Tc) 12,5 μg/ml.

Liquid cultures were inoculated with a 1:1.000 dilution (when cultures were grown at 37 °C) or 1:100 (when cultures were grown at 25 °C) from a previous overnight culture (16 h at 37 °C) in LB with vigorous shaking at 200 rpm.

### Genetic manipulations and plasmids construction

Mutant derivatives lacking the *hns* paralogue genes in the EAEC strain 042 were obtained by the λ Red recombinant method previously described^33^. Briefly, the antibiotic resistance determinant of the plasmid pKD4 (kanamycin) was amplified using the corresponding oligonucleotides hns p1/hns p2, hns2 p1/hns2 p2 and stpA p1/stpA p2 for *hns*, *hns2* and *stpA* genes respectively (Supplementary Table S2). The mutants were confirmed by PCR using the KT primer (located inside the kanamycin resistance) in combination with specific primers that hybridizes in the remaining gene sequence in the bacterial chromosome (hnsp1 up for *hns*, hns2p1 up for *hns2* and stpA p1 up for *stpA*), respectively (Supplementary Table S2). When necessary, the antibiotic resistance determinant was eliminated by using the FLP/FRT-mediated site-specific recombination method, as previously described^34^. The double mutants were obtained by combining a previous deletion with another deletion generated by insertion of another antibiotic resistance cassette.

Recombinational transfer of the Flag-epitope into the C-terminal end of the *hns2* gene was achieved by following the methodology previously described^35^. The template vector coding for the Flag-epitope and Km^r^ determinant used was pSUB11. The oligonucleotides used for the construction of the H-NS2 Flag-tagged derivative were hns23X p1 and hns23X p2 (Supplementary Table S2). The correct insertion of the Flag-tag was confirmed by PCR using the oligonucleotides hns2 p1 up/hns2 p2 down (Supplementary Table S2).

The *hns2* gene of *E*. *coli* 042 with a point mutation on its own promoter (clone obtained in the directed evolution experiment) was cloned into the low copy number pLG338-30 plasmid. Oligonucleotides hns2pLG338 ECORI fw 5 and hns2pLG338 BAMHI rev3 (Supplementary Table S2) were used to amplify by PCR the *hns2* gene using the *Phusion Hot Start II DNA polymerase* (*Thermo Scientific*) following the manufacture´s recommendations. The resulting PCR fragment was purified using the *GeneJET PCR purification Kit* (*Thermo Scientific*) and digested with EcoRI and BamHI restriction enzymes (*Thermo Scientific*). Ligation was performed with pLG338-30 plasmid digested with the same restriction enzymes and treated with alkaline phosphatase (*FastAP*, *Thermo Scientific*). The resulting recombinant plasmid pLGhns2* was transformed in *E*. *coli* MG1655 cells. Plasmid extraction was carried out from positive clones using *GeneJET Plasmid Miniprep Kit* (*Thermo Scientific*) and subsequently Sanger sequenced for confirmation of correct in-frame insertion of the *hns2* gene using the primers pLG338 EB Fw and pLG338 EB Rv (Supplementary Table S2).

For construction of transcriptional *lacZ* fusions with the *hns2* promoter, the *hns2* promoter region from *E*. *coli* 042 was PCR amplified by using the *Phusion Hot Start II DNA Polymerase* (*Thermo Scientific*), following the manufacturer´s recommendations, using the primers *hns2* promoter Fw EcoRI/hns2 promoter Rev BamHI (Supplementary Table S2) and using as a templates the *E*. *coli* 042 wild type strain and the spontaneous mutant *E*. *coli* 042 Δ*hns* with the C > T transition generated in the directed evolution experiment. The resulting PCR fragments were purified by using the *GeneJET PCR purification Kit* (*Thermo Scientific*) and digested with EcoRI and HindIII restriction enzymes (*Thermo Scientific*). Ligation was performed in the low copy plasmid pFZY-1 digested with the same restriction enzymes and treated with alkaline phosphatase (FastAP, *Thermo Scientific*). The resulting plasmids pFZY1*phns2* (wild type *hns2* promoter) and pFZY1phns2* (*hns2* promoter with the C > T change) were transformed in *E*. *coli* MG1655 cells. Plasmid DNA was extracted from the transformants and purified by using the *GeneJET PCR purification Kit* (*Thermo Scientific*) and Sanger sequenced for confirmation of the correct in-frame insertion of the *hns2* promoter by using the primers pFZY-1 p1up-lacZ Rv (Supplementary Table S2).

To introduce plasmids in *E*. *coli*, bacterial cells were grown until an optical density at 600 nm (O.D_600nm_) of 0.6–0.8. Once cells arrived at this level of growth, they were washed several times with 10% glycerol at 4 °C, and the respective plasmids were introduced by electroporation using an *Eppendorf* gene pulser (*Electroporator 2510*).

### Whole genome sequencing and analysis

Short-read genome sequencing was performed by using the *Illumina* technology. DNA was isolated from overnight cultures using the *NucleoMag 96 Tissue Kit* (*Macherey–Nagel*, Düren, Germany). Sequencing libraries were prepared by using the Nextera XT *Kit* (*Illumina*, Eindhoven, Netherlands). Sequencing was performed on a *NextSeq* 500 machine with 2 × 150 nt read length. An average read length of 117.8 nt and a coverage of 94 × was achieved. Read mapping of the reads was performed using the *ASA*^*3*^*P* and *CLC Genomics Workbench* (v. XXX, *Qiagen*, Hilden, Germany). Analysis of the Single nucleotide polymorphisms (SNPs) and deletions was performed using *ReadXplorer v*.*2*.*1*.*0* and *CLC Genomics Workbench*. Regions not covered were considered as deleted regions.

### Directed evolution protocol

The *E*. *coli* 042 Δ*hns* stock mutant stored at -80 °C was spread over an LB agar plate and incubated overnight at 37 °C. Six independently derived colonies were selected, and each of these Δ*hns* mutant single colonies was used to inoculate 10 ml of fresh LB medium (three out of six were incubated at 25 °C and the rest at 37 °C). Each overnight culture (day 0) was used as the starter culture for a series of 10-days subcultures in 25 ml of LB medium using as an inoculum the overnight culture from the previous day (with a dilution of 1:1.000 for incubation at 37 °C and 1:100 for incubation at 25 °C). At days 1, 5 and 10 of subcultures, bacterial growth was monitored in all cultures by measuring the optical density at 600 nm. Aliquots of each daily culture were used for PCR amplification and subsequent sequencing of the *hns2* and *stpA* genes. In addition, 1.5 ml samples from each culture were freezed at -80 °C.

### Electrophoresis and western blotting analysis

Whole-cell protein extracts were prepared in Laemmli buffer^36^ (glycerol 5% v/v, β-mercaptoethanol 2.5%, SDS 1.15% p/v, Tris–HCl 31 mM pH 6.6 and bromophenol blue 0.05%). Protein samples were analyzed by Tris-Glicine-SDS triphasic gels with 16.5% polyacrylamide. Proteins were transferred from the gels to PVDF membranes by using the semidry electrophoretic transfer cell (*Bio-Rad*) at 15 V during 40 min. For the Western blot analysis, a monoclonal antibody directed against the Flag-epitope (*Sigma-Aldrich*) diluted 1:10.000 in a solution of PBS, 0.2% Triton and 3% skimmed milk was used. The membranes containing the proteins were incubated with the diluted antibody for 16 h at 4 °C. The membranes were afterwards washed for 10 min with PBS, 0.2% Triton X-100 (*Sigma-Aldrich*) solution. The washing step was repeated three times. Thereafter, the membranes were incubated with horseradish peroxidase-conjugated goat anti-mouse IgG (*Promega*) diluted 1:2.500 in a solution of PBS, 0.2% Triton X-100 for 45 min at room temperature. Again, three washing steps of 30 min with PBS, 0.2% Triton solution were performed, and immunodetection of the specific protein was performed by enhanced chemiluminescence by using the *Molecular Imager ChemiDoc XRS* system and *Quantity One* software (*Bio-Rad*).

### β-galactosidase assay

β-galactosidase activity assays were performed as described previously^37^. Student´s *t*-test was used to determine the statistical significance, and the values were obtained by using the GraphPad Prism 5 software. A *P* value of less than 0.05 was considered significant.

### RNA-Seq

RNA-seq experiments were performed as previously described^28^.

### Isolation of RNA

For RNA isolation, bacterial cells were grown until OD_600_ of 2.0. Then, 5 ml of cells were mixed with a 0.2 × volume of a stop solution (95% ethanol, 5% phenol), shaken and centrifuged during 10 min at 6.000 × g. Bacterial pellets were subsequently frozen at -80 °C until use. Total RNA was extracted from bacterial pellets using *Tripure Isolation Reagent* (*Roche*) according to the manufacturer’s instructions. Potential traces of DNA were removed by digestion with *AmbionDNase I (RNase-free)* according to the manufacturer’s instructions. RNA concentration and quality were measured using a Nano-Drop 1000 (*Thermo Scientific*).

### 5′ RACE (Rapid amplification of cDNA ends)

5´ RACE assays were performed for the determination of the 5´-mRNA transcript of *hns2* gene from strains 042 WT strain and its Δ*hns* and Δhns hns2* derivatives. The assays were carried out essentially as reported^38^, with minor modifications as described. The 5´ triphosphates were converted to monophosphates by treatment of 12 µg of total RNA with 25 units of tobacco acid pyrophosphatase (*TAP*, *Epicentre*), 1 × final concentration of *TAP* buffer plus 0.5 µl of *RNase Inhibitor* (*Ambion*) during 1 h at 37 °C. Control RNA was incubated under the same conditions in the absence of the TAP enzyme. Before P:C:I (water-saturated phenol, chloroform, isoamyl alcohol mixture, 25:24:1, v/v) extraction, 500 pmol of the RNA adapter oligonucleotide A3 (Supplementary Table S2) were added to both reactions. After P:C:I extraction, RNAs were ethanol precipitated in the presence of 0.3 M sodium acetate (pH 5.7). RNAs were dissolved in water, heat-denatured at 90 °C for 5 min, and then quick-chilled on ice. The A3 adapter was ligated at 17 °C for 16 h by using *T4 RNA ligase* (*Thermo Scientific*) plus *RNase Inhibitor* (*Ambion*) pre-mixed in a ratio of 9:1 (RNA ligase:RNase Inhibitor), *T4 RNA ligase* buffer (*Thermo Scientific*) and 10% of DMSO. RNAs ligated to A3 oligonucleotide were then P:C:I purified and ethanol-precipitated. RNAs were redissolved in water, and 5 µg were reverse transcribed by using random primers and the *High-capacity cDNA Reverse Transcription Kit* (*Applied Biosystems*) according to the manufacturer’s instructions. Reverse transcription was performed in three cycles of: 10 min at 25 °C, 120 min at 37 °C and 5 min at 85 °C. The products of reverse transcription were amplified by using the *DreamTaq Hot Start Green DNA Polymerase* (*Thermo Scientific*). For each 50 µl of PCR reaction, 2 µl of cDNA were used together with 0.5 µM of the *hns2* gene specific (hns2-RACE Rv) and adapter specific (B6) primers (Supplementary Table S2). PCR products were separated on 2% agarose gels, and the single-band amplicons were purified by using the *GeneJET PCR purification Kit* (*Thermo Scientific*) and cloned into pCR2.1TOPO (*Invitrogen*) vectors following the manufacture´s recommendations. White colonies were screened for the presence of inserts of appropriate size by colony PCR using M13 Fw and M13 Rv primers (Supplementary Table S2), and Sanger sequenced using the same M13 primers.

### Quantitative reverse transcription-PCR (qRT-PCR)

The expression level of the *hdeA*, EC042_RS01720, EC042_RS23490, *slp* and *hcp* genes was determined by using real-time quantitative PCR. Briefly, 1 μg of previously isolated total RNA was reverse transcribed to generate cDNA by using a *High-capacity cDNA Reverse Transcription Kit* (*Applied Biosystems*) according to the manufacturer’s instructions. All samples within an experiment were reverse transcribed at the same time. The resulting cDNA was diluted (1:100) in nuclease-free water and stored in aliquots at –80 °C until used. As a control, parallel samples in which reverse transcriptase was omitted from the reaction mixture were run. Real-time PCR was carried out by using the *Maxima SYBR green/ROX qPCR Master Mix* (*Thermo Scientific*) and an *ABI Prism 7700* sequence detection system (*Applied Biosystems*). Specific oligonucleotides complementary to the genes of interest were designed by using the *Primer3* software (Supplementary Table S2). The relative quantification of gene expression of mutants versus the wild-type strain was performed using the comparative threshold cycle (CT) method^39^. The relative amount of target cDNA was normalized by using the *gapA* gene as an internal reference standard. Student´s *t*-test was used to determine the statistical significance, and the values were obtained by using the GraphPad Prism 5 software. A *P* value of less than 0.05 was considered significant.

### Bioinformatics analysis

UniProt (https://www.uniprot.org) accession numbers of differentially expressed genes were used to annotate the Gene Ontology (GO) terms. For the grouping of genes differentially expressed by categories a Python script was programmed^40,41^.

The analysis of the *hns2* promoter prediction was performed using the BPROM software (https://www.softberry.com/berry.phtml?topic=bprom&group=programs&subgroup=gfindb).

### Data availability

The RNA sequencing reads have been deposited in the Gene Expression Omnibus (GEO) Sequence Read Archive of the National Center for Biotechnology Information (GSE146396), under accession numbers GSM4382146, GSM4382147 and GSM4382148, for experiments performed at 25 °C. Experiments performed at 37 °C, previously published^28^, reads were already deposited in GEO (GSE105133), under accession numbers GSM2822965, GSM2822967 GSM2822968.

Raw data generated from whole genome sequencing experiment are available on Sequence Read Archive (SRA) under the BioProject ID PRJNA631595.

## Supplementary information

Supplementary material 1
