# Supplementary material for: Overexpression of the third H-NS paralogue H-NS2 compensates fitness loss in hns mutants of the enteroaggregative Escherichia coli strain 042
Source: Sci Rep. 2020 Oct 22;10:18131. doi: 10.1038/s41598-020-75204-4 (PMC7582179; doi:10.1038/s41598-020-75204-4)
Supplement: Supplementary file 1 — Supplementary material 1 [file 41598_2020_75204_MOESM1_ESM.docx]

**Supplementary Information (tables, figures and blot images)**

**Overexpression of the third H-NS paralogue H-NS2 compensates fitness loss in *hns* mutants of the enteroaggregative *Escherichia coli* strain 042**

Prieto, A.^1^, Bernabeu, M.^1^, Falgenhauer, L.^2,3^, Chakraborty, T.^3,4^, Hüttener, M.^1^ and Juárez, A.^1,5*^

^1^Department of Genetics, Microbiology and Statistics, University of Barcelona, Barcelona, Spain.

^2^Institute of Hygiene and Environmental Medicine, Justus Liebig University Giessen, Schubertstrasse 81, 35392 Giessen, Germany.

^3^German Center for Infection Research DZIF, Partner site Giessen-Marburg-Langen, Campus Giessen, Germany.

^4^Institute of Medical Microbiology, Justus-Liebig University, Schubertstrasse 81, 35392 Giessen, Germany.

^5^Institute for Bioengineering of Catalonia (IBEC), The Barcelona Institute of Science and Technology, Barcelona, Spain.

*Corresponding author: Prof. Antonio Juárez. [ajuarez@ub.edu](mailto:ajuarez@ub.edu)

**Supplementary Table S1.** Bacterial strains and plasmids used in this work.

| Bacterial strain | Description | Source/Reference |
| --- | --- | --- |
| *E. coli* 042 WT | *E. coli* EAEC, Cm^r^ Sm^r^ Tc^r^ | Prof. I. Henderson |
| *E. coli* 042 Δ*lacZ* | 042 derivative carrying *lacZ* gene deletion | This work |
| *E. coli* 042 Δ*hns* | 042 derivative carrying *hns* deletion. | ^1^ |
| *E. coli* 042 Δ*stpA* | 042 derivative carrying *stpA* deletion. | This work |
| *E. coli* 042 Δ*hns2* | 042 derivative carrying *hns2* deletion. | ^2^ |
| *E. coli* 042 Δ*hns*Δ*hns2* | 042 derivative carrying *hns and hns2* deletion. | ^2^ |
| *E. coli* 042 Δ*hns*Δ Δ*stpA* | 042 derivative carrying *hns* and *stpA* deletion. | This work |
| *E. coli* 042 Δ*hns phns2** | 042 derivative carrying *hns* deletion with the transition C>T generated in the -10 box of *hns2* promoter. | This work |
| *E. coli* 042 *hns2*-FLAG | 042 derivative carrying epitope FLAG added to *hns2* C-terminal. | ^2^ |
| *E. coli* 042 Δ*hns hns2-FLAG* | *E. coli* 042 Δ*hns* derivative carrying epitope FLAG added to *hns2* C-terminal. | This work |
| *E. coli* 042 Δ*hns phns2* hns2-FLAG* | *E. coli* 042 Δ*hns* with the transition C>T in -10 box of *hns2* promoter with epitope FLAG added to *hns2* C-terminal. | This work |
| *E. coli* 042 Δ*lacZ* Δ*cpxR* | 042 derivative carrying *lacZ* and *cpxR* deletion | This work |
| *E. coli* MG1655 | *E. coli* K12 substr. MG1655. | Lab stock |
| *E. coli* DH5α | *Φ80dlacZΔM15 recA1 endA1 gyrA96 thi1 hsdR17 (rk- mk+) supE44 relA1 deoRΔ(lacZYA-argF)U169 phoA.* | ^3^ |
| Plasmid | **Description** | **Source/Reference** |
| pKD4 | *bla* (Ap^r^) FRT *ahp* FRT PS1 PS2 oriR6K Km^r^ | ^4^ |
| pKD46 | *bla* (Ap^r^) P_BAD_ *gam bet exo* pSC101 oriTS | ^4^ |
| pSUB11 | R6KoriV -FLAG and -Km^r^ coding template vector | ^5^ |
| pCP20 | *bla* (Cb^r^) *cat* λ *cI*857 *l*P_R_*flp* pSC101 oriTS | ^4^ |
| pLG338-30 | OripSC101, Ap^r^ | ^6^ |
| pLG338-30*hns2* | pLG338-30 + *hns2* gene from *E. coli* 042 | ^2^ |
| pLG338-30*hns2** | pLG338-30 + *hns2* gene from *E. coli* 042 with transition C>T generated in the -10 box of *hns2* promoter | This work |
| pFZY1 | F ́ *lac* replicon *lacZYA*+ Ap^r^ | ^7^ |
| pFZY1*phns2* | *hns2* WT promoter cloned in pFZY-1 plasmid | This work |
| pFZY1*phns2** | *hns2* with transition C>T in the -10 box of *hns2* promoter cloned in pFZY-1 plasmid | This work |

**Supplementary Table S2.** Oligonucleotides used in this work.

| Oligonucleotide name | Sequence (5’-3’) | Use |
| --- | --- | --- |
| hns p1 | ATGAGCGAAGCACTTAAAATTCTGAACAACATCCGTACTCTTGTGTAGGCTGGAGCTGCTTC | *hns* deletion |
| hns p2 | TTATTGCTTGATCAGGAAATCGTCGAGGGAT TACCTTGCTGCATATGAATATCCTCCTTAGT | *hns* deletion |
| hns p1 up | CCTCACGTGCTGCGAAATCA | *hns* deletion confirmation |
| hns p2 down | GCAGGCCTTCGTTGAATACG | *hns* deletion confirmation |
| hns2 p1 | GCTTGTATTCTTTACCACAAACATTAGGGAAATCGCATGTCTGTGTAGGCTGGAGCTGCTTC | *hns2* deletion |
| hns2 p2 | AAAATGAGCGGATTTGGTAGACATATGGTATGAAGATTACTTCATATGAATATCCTCCTTAGT | *hns2* deletion |
| hns2 p1 up | TAAATTTGAACTCAGGATGGA | *hns2* deletion confirmation and gene sequencing |
| hns2 p2 down | TGGCATGTTAATCCCTGTAGG | *hns2* deletion confirmation and gene sequencing |
| stpA p1 | TTTTTGTTTTGGCGTTAAAAGGTTTTCTTTATTATGTCCGTAGTGTAGGCTGGAGCTGCTTC | *stpA* deletion |
| stpA p2 | ACGCGCCATAGCAGCGACATCCGGCCTCAGTAATTAGATCAGCATATGAATATCCTCCTTAGT | *stpA* deletion |
| stpA p1 up | GGAATTAGCGAGCAGAGAGCG | *stpA* deletion confirmation and gene sequencing |
| stpA p2 down | ACGCACCAGTAATAACGCTGA | *stpA* deletion confirmation and gene sequencing |
| hns23x p1 | GCTCGAAGCCGGTAAGACACTGGAAGATTTCGAGATCAAGGACTACAAAGACCATGACGG | *hns2* FLAG insertion |
| hns23x p2 | ATGAGCGGATTTGGTAGACATATGGTATGAAGATTACTTGCATATGAATATCCTCCTTAG | *hns2* FLAG insertion |
| KT | CGGCCACAGTCGATGAATCC | Km cassette insertion confirmation |
| hns2 pLG338 ECORI fw 5 | CGGAATTCTAAATTTGAACTCAGGATGGA | *hns2* cloning in pLG338 vector |
| hns2 pLG338 BAMHI rev 3 | CGGGATCCTGGCATGTTAATCCCTGTAGG | *hns2* cloning in pLG338 vector |
| pLG338 EB Fw | CCGCGCACATTTCCCCGAAAA | Confirmation of *hns2* cloning into pLG338-30 vector |
| pLG338 EB Rv | GCCAGTGCCAAGCTAGGCCTA | Confirmation of *hns2* cloning into pLG338-30 vector |
| hns2 promoter Fw EcoRI | CGGAATTCTAAATTTGAACTCAGGATGGA | Cloning of *hns2* promoter in pFZY1 vector |
| hns2 promoter Rev BamHI | CGGGATCCGCGATTTCCCTAATGTTTGTGG | Cloning *hns2* promoter in pFZY1 vector |
| pFZY1 p1up | CCATTATTATCATGACATTAA | Confirmation of *hns2* promoter cloning into pFZY1 |
| lacZ Rv | GATGACCTGCAAGGCGATTA | Confirmation of cloning into pFZY-1 |
| gapA Fw | TTTCCGTGCTGCTCAGAAAC | qPCR |
| gapA Rv | GTCAACACCAACTTCGTCCC | qPCR |
| A3 | GAUAUGGCGGAAUUCCUGUAGAACGAACACUAGAAGAAA | *hns2* 5’RACE oligonucleotide adapter |
| B6 | GCGCGAATTCCTGTAGA | *hns2* 5’RACE PCR confirmation |
| hns2-RACE Rv | TCCACGTCTTTTCCGAACCA | *hns2* 5’RACE PCR confirmation |
| M13 Fw | GTTTTCCCAGTCACGAC | Sequencing |
| M13 Rv | CAGGAAACAGCTATGAC | Sequencing |
| hdeA RT Fw | GGACGAATCCTTCCAGCCAA | qPCR |
| hdeA RT Rv | CGATAGCTGGGGTTACGGTT | qPCR |
| RS01720 RT Fw | ACTACCGCTTTCAGTACGCC | qPCR |
| RS01720 RT Rv | TCCTTTGTTATCCGCCGTGT | qPCR |
| RS23490 RT Fw | GCAGCAACGTCGGTTTCG | qPCR |
| RS23490 RT Rv | GGTCGTGATTGTTGTGGTCC | qPCR |
| slp RT Fw | TTTGCTTGCCGCATGTAGTT | qPCR |
| slp RT Rv | ACGTTGATAACCTTCCCGCC | qPCR |
| Hcp RT Fw | CCGATCCACAGTCTGGTCAA | qPCR |
| Hcp RT Rv | CCACTTTCGGCAGCATTTCA | qPCR |

**REFERENCES**

1.	Hüttener, M., Dietrich, M., Paytubi, S. & Juárez, A. HilA-like regulators in *Escherichia coli* pathotypes: the YgeH protein from the enteroaggregative strain 042. *BMC Microbiol.* **14,** 268 (2014).

2.	Prieto, A. *et al.* Evolution of Bacterial Global Modulators: Role of a Novel H-NS Paralogue in the Enteroaggregative *Escherichia coli* Strain 042. *mSystems* **3,** (2018).

3.	Taylor, R. G., Walker, D. C. & McInnes, R. R. *E. coli* host strains significantly affect the quality of small scale plasmid DNA preparations used for sequencing. *Nucleic Acids Res.* **21,** 1677–1678 (1993).

4.	Datsenko, K. A. & Wanner, B. L. One-step inactivation of chromosomal genes in *Escherichia coli* K-12 using PCR products. *Proc. Natl. Acad. Sci. U.S.A.* **97,** 6640–6645 (2000).

5.	Uzzau, S., Figueroa-Bossi, N., Rubino, S. & Bossi, L. Epitope tagging of chromosomal genes in *Salmonella*. *Proc. Natl. Acad. Sci. U.S.A.* **98,** 15264–15269 (2001).

6.	Cunningham, T. P., Montelaro, R. C. & Rushlow, K. E. Lentivirus envelope sequences and pro viral genomes are stabilized in *Escherichia coli* when cloned in low-copy-number plasmid vectors. *Gene* **124,** 93–98 (1993).

7.	Koop, A. H., Hartley, M. E. & Bourgeois, S. A low-copy-number vector utilizing beta-galactosidase for the analysis of gene control elements. *Gene* **52,** 245–256 (1987).

**Supplementary Figure S1.** Determination by 5’ RACE of the transcriptional start site (+1) of the *hns*2 gene in the strain 042 WT, and in its *hns* and *hns2** derivatives.

**Supplementary Figure S2.** Bioinformatic analysis of the *hns2* regulatory and coding sequences, both in the WT and in the *phns2** mutant backgrounds. -35 and -10 regions, as well as the start codon are boxed. The putative CpxR binding sequence is highlighted in yellow.

**Supplementary Figure S3.** Expression of transcriptional *hns2::lacZ* and *hns2*::lacZ* fusions in the 042 WT strain and in its *cpxR* mutant derivative. The bar shows the arithmetic mean of the results of three independent experiments and the error bar indicates the standard deviation. Statistical analysis showed a significant difference (*P-value < 0.0001, **P-value < 0.0331, ***P-value < 0.0024).

**Supplementary Figure S4A.** PCR amplification of the *hns2* gene in strains 042 WT and its *hns2, hns2*, hns* (pLG*hns2*) and *hns* (pLG*hns2**) derivatives.

**Supplementary Figure S4B.** Sequence of the IS*5* inserted (in red) in the coding region of the *hns2* gene cloned in the pLG338-30 vector under the control of the *phns2** promoter.

**Supplementary Figure S5.** Functional categories of the up- and downregulated genes in strains 042 *hns* and 042 *hns2*, when compared to the WT strain. Cultures were grown either at 25ºC (A) or 37ºC (B).

**Images of western blot analyses – Manuscript**

**Figure 3B** – coomassie blue gels

**Figure 3B** – western blot
